# Supplementary material for: Off the shelf: Investigating transfer of learning using commercially available virtual reality equipment
Source: PLoS One. 2023 Oct 3;18(10):e0279856. doi: 10.1371/journal.pone.0279856 (PMC10547175; doi:10.1371/journal.pone.0279856)
Supplement: Attachment — Submitted filename: Responses to Reviewers.docx [file pone.0279856.s002.docx]

PONE-D-22-34509

Off the shelf: Investigating transfer of learning using commercially available virtual reality equipment

PLOS ONE

Dear Dr. Markwell,

Thank you for submitting your manuscript to PLOS ONE. After careful consideration, we feel that it has merit but does not fully meet PLOS ONE’s publication criteria as it currently stands. Therefore, we invite you to submit a revised version of the manuscript that addresses the points raised during the review process.

Please submit your revised manuscript by Jul 22 2023 11:59PM. If you will need more time than this to complete your revisions, please reply to this message or contact the journal office at [plosone@plos.org](mailto:plosone@plos.org). When you're ready to submit your revision, log on to https://www.editorialmanager.com/pone/ and select the 'Submissions Needing Revision' folder to locate your manuscript file.

Please include the following items when submitting your revised manuscript:

- A rebuttal letter that responds to each point raised by the academic editor and reviewer(s). You should upload this letter as a separate file labeled 'Response to Reviewers'.
- A marked-up copy of your manuscript that highlights changes made to the original version. You should upload this as a separate file labeled 'Revised Manuscript with Track Changes'.
- An unmarked version of your revised paper without tracked changes. You should upload this as a separate file labeled 'Manuscript'.

If you would like to make changes to your financial disclosure, please include your updated statement in your cover letter. Guidelines for resubmitting your figure files are available below the reviewer comments at the end of this letter.

If applicable, we recommend that you deposit your laboratory protocols in protocols.io to enhance the reproducibility of your results. Protocols.io assigns your protocol its own identifier (DOI) so that it can be cited independently in the future. For instructions see: <https://journals.plos.org/plosone/s/submission-guidelines#loc-laboratory-protocols>. Additionally, PLOS ONE offers an option for publishing peer-reviewed Lab Protocol articles, which describe protocols hosted on protocols.io. Read more information on sharing protocols at <https://plos.org/protocols?utm_medium=editorial-email&utm_source=authorletters&utm_campaign=protocols>.

We look forward to receiving your revised manuscript.

Kind regards,

Nick Fogt
Academic Editor
PLOS ONE

Journal Requirements:

When submitting your revision, we need you to address these additional requirements.

1. Please ensure that your manuscript meets PLOS ONE's style requirements, including those for file naming. The PLOS ONE style templates can be found at 
https://journals.plos.org/plosone/s/file?id=wjVg/PLOSOne_formatting_sample_main_body.pdf and 
https://journals.plos.org/plosone/s/file?id=ba62/PLOSOne_formatting_sample_title_authors_affiliations.pdf

2. Please provide additional details regarding ethical approval in the body of your manuscript. In the Methods section, please ensure that you have specified the name of the IRB/ethics committee that approved your study.

3. Please ensure that you include a title page within your main document. You should list all authors and all affiliations as per our author instructions and clearly indicate the corresponding author.

4. Please include captions for your Supporting Information files at the end of your manuscript, and update any in-text citations to match accordingly. Please see our Supporting Information guidelines for more information: http://journals.plos.org/plosone/s/supporting-information. 

Additional Editor Comments:

This paper addresses an important question regarding virtual reality training and motor transfer. Of particular concern here is Reviewer #1s and Reviewer #2s comments regarding the statistical analyses and the appropriateness of the study design in terms of directly addressing the question of transfer the authors sought to answer. These comments from both reviewers should be carefully addressed. The authors should also comment on the advantages of Reviewer #1s proposal of analyzing changes (perhaps on an individual basis) within the training environment (eg. virtual training with outcomes in virtual reality) in addition to the transfer to real-world conditions. In other words, is there something to be learned by examining the changes that occur within each environment? Of perhaps most concern is Reviewer #1s comment on the time course of the study. Can the authors provide some reasoning or evidence as to whether such a short period of training and testing is adequate to support the authors' conclusions?

**Response**: Thank you for taking the time to review this manuscript. We have responses to each of the reviewers’ comments.

Regarding the statistical analysis comment (Reviewer #1, comment #3), the authors are not exactly sure what reviewer #1 is suggesting. In the present study, test 3 was performed at least 24 hours after the end of the acquisition phase. It was used as a delayed retention test for the RW group and a delayed transfer test for the VR group. The design of this test aligns with a common practices when assessing motor learning (Magill & Anderson, 2020; Schmidt et al., 2018). Additionally, since both practice groups showed performance improvements from day 1 to day 2, this would suggest that there was an adequate number of practice trials to assess motor learning. Regarding the analysis, a mixed repeated measures ANOVA has been frequently used to compare motor learning differences between two practice groups. More specifically, this form of analysis is consistent with previous VR research which is cited in our manuscript. We acknowledge that we might not have clarified this well within the original manuscript. Revisions have been made in hopes to clarify both the design and analysis.

Lastly, the introduction should be edited for content and length as suggested by Reviewer #1, and the discussion should center around how the results relate to theories (eg. identical elements theory) discussed in the introduction.
**Response**: We have cut out numerous sections to streamline the introduction. Additionally, a section has been added to the discussion relating the results to the theories mentioned.

[Note: HTML markup is below. Please do not edit.]

Reviewers' comments:

Reviewer's Responses to Questions

**Comments to the Author**

1. Is the manuscript technically sound, and do the data support the conclusions?

The manuscript must describe a technically sound piece of scientific research with data that supports the conclusions. Experiments must have been conducted rigorously, with appropriate controls, replication, and sample sizes. The conclusions must be drawn appropriately based on the data presented.

Reviewer #1: No

Reviewer #2: Yes

2. Has the statistical analysis been performed appropriately and rigorously?

Reviewer #1: No

Reviewer #2: Yes

3. Have the authors made all data underlying the findings in their manuscript fully available?

The [PLOS Data policy](http://www.plosone.org/static/policies.action#sharing) requires authors to make all data underlying the findings described in their manuscript fully available without restriction, with rare exception (please refer to the Data Availability Statement in the manuscript PDF file). The data should be provided as part of the manuscript or its supporting information, or deposited to a public repository. For example, in addition to summary statistics, the data points behind means, medians and variance measures should be available. If there are restrictions on publicly sharing data—e.g. participant privacy or use of data from a third party—those must be specified.

Reviewer #1: Yes

Reviewer #2: Yes

4. Is the manuscript presented in an intelligible fashion and written in standard English?

PLOS ONE does not copyedit accepted manuscripts, so the language in submitted articles must be clear, correct, and unambiguous. Any typographical or grammatical errors should be corrected at revision, so please note any specific errors here.

Reviewer #1: No

Reviewer #2: Yes

5. Review Comments to the Author

Please use the space provided to explain your answers to the questions above. You may also include additional comments for the author, including concerns about dual publication, research ethics, or publication ethics. (Please upload your review as an attachment if it exceeds 20,000 characters)

Reviewer #1: This is an interesting article regarding motor learning and transfer, however, there are some concerns related to the organization of the manuscript, methods and statistical analisys. Which made me assume, that this kind of work need to be better strutured.

**Response**: Thank you for your interest and your time reviewing our manuscript. We have made significant revisions based on your recommendations.

1. Extremely extensive introduction, with moments that should be placed in the discussion sector and not introduction.
I believe that better organization of this part is necessary, for a more fluid and better organized line of reasoning on the subject in question. Due to the amount of information, it becomes tiring, extensive and the central objective of the work is confusing.

**Response**: Thank you for this suggestion. The information within the introduction has been reduced. Information within the first and second paragraph has been omitted in hopes to streamline the most relevant information and problem.

2. Statistical analysis, poorly organized and poorly described.
How was the homogeneity test performed?
How was the data distribution verified so that the ANOVA could be performed?
What were the kinetic variables evaluated?
The information is scattered throughout the text, in the section where only the results should be written.

**Response:** The homogeneity of variances was examined using Levene’s test. It did not reveal evidence of significant differences between groups, indicating the assumption of homogeneity were met. Sphericity was examined using Mauchly’s test of sphericity. When significant, this was listed in the result section. This information has been added to the statistical analysis section. No kinetic variables were evaluated. As mentioned in the data analysis (lines 181-183) and listed in the results section, the four dependent variables that were analyzed were accuracy (performance outcome) and three performance production (kinematic) variables: backswing displacement, follow-through displacement, and club head velocity. Subheadings have been edited for clarity.

3. The article presents problems of statistical analysis and methods that are concerning. If transfer was what was being analyzed, it was not necessary to perform a repeated measures analysis considering interaction factors between and within groups. Only a pre and post test analysis comparing the practice groups was enough.
And actually, what is being compared are the differences between practice groups and not the transfer of learning itself.
Also, it takes more practice time and more retention analysis time to verify that the individual has really learned. Remembering that learning translates into continuous changes in behavior over time, with at least 24 hours of a good night's sleep so that there is retention and, after this the transfer proposal.
Information about the study participants is not clearly found, which further mades more bias for the data.
A more correct pilot study, to later carry out a transfer study, would be with two practice groups: virtual reality and real world, and the practice observations of each group, with the learning curves of each individual.
The virtual reality group practices and is analyzed through virtual reality.
The real world group practices and is analyzed by the real world.
The learning curves are verified and then comparisons between groups from the learning results, taking into account the practice.
And later on, a proposal for learning transfer.
It is not even known correctly whether this practice of golf in virtual reality is good enough to be compared with the real world.

**Response**: The authors are unclear what reviewer #1 is exactly proposing for the analysis in comment #3. However, we acknowledge that performance differences between practice groups is what is being assessed. Specifically, retention is being assessed for the real-world group and transfer is being assessed for the VR group, given that the post tests were performed in the real-world environment. Additionally, we acknowledge that it is important to assess retention after a minimum of 24 hours. This is why test 3 was performed. It acts as a 24 hour retention test for the real-world group and a 24 hour transfer test for the VR group. This is mentioned on lines 173-175. While there have been VR studies that have only used t-test to examine post test differences, there are also other studies that have used between-within analyses. Furthermore, by assessing the between-within differences after a minimum of 24 hours following the acquisition period, our analyses align with well-established criteria for assessing motor learning and transfer (Gray, 2019; Magill, 2020; Schmidt et al., 2019).

4. Which is the importance of this practice? Why was golf chosen? This is not clear.

**Response**: A golf putting task what chosen because it has been a commonly used task to assess motor learning (Daou et al., 2016, 2019; McKay et al., 2021 Salehi et al., 2021), and specifically for motor learning in VR (Harris et al., 2020; Markwell et al., in press). Because it has been frequently used, the number of practice trials performed were expected to facilitate motor learning based on the performance outcomes during a 24 hour retention test. Furthermore, given the purpose of the study, it was important to choose a task that is commercially available for the VR practice. Lastly, golf putting provides logistical benefits as it can be recreated in a controlled laboratory environment. This rationale has been included in the method section of our revised manuscript.

Reviewer #2: In this article the authors sought to investigate whether training a mechanical task (i.e., swinging a golf club for putting) in virtual reality (VR) transfers to the real world (RW) and how this virtual training compares to real-world training. The authors designed and empirical study in which the experiment took place over two days. On the first day, participants were tasked with completing a pre-test in which they putted a golf ball 10 times in the real world, followed by a 60 practice putts in either a VR or RW training session, followed by a post-test task of 10 additional putts in the real world. This procedure was then repeated on the second day. Data was recorded for accurary of the putts, as well as kinematic measurse of club backswing, follow-through and velocity. The authors anticipated that both groups would improve in putting performance, with no significant difference between the VR and RW training groups. Further, they anticipated that there would be no significant difference between the two groups in terms of kinematic performances. The data appear to generally support these hypotheses.
Overall, this paper adds to the limitted existing literature that examines how commercially available VR programs can be used for training mechanical tasks. These findings are important as they provide additional evidence that VR training may be comparable to real-world training. Given the wide range of diciplines that are attempting to use VR for training purposes, this research is both very relevant and impactful. Recommendation is to accept, provided authors address some minor issues.

**Response**: Thank you for your time reviewing this article and the recommendations. You have provided valuable feedback. We have made significant revisions based on your comments and we feel that we have used this feedback to strengthen our manuscript.

Introduction
• While the previous work of Michalski et al. and Drew et al. were included, it appears that the respresentation of Drew et al.’s findings are slightly misleading. In line 122-123 it is stated that Drew et al.’s findings did not demonstrate kinematic differences during post test; while correct, it should be noted that kinematic differences were observed during training, and that participants appeared to adjust their methods during the post-test task.

**Response**: Thank you for this comment. Our intent is not to mislead the reader about Drew et al.’s findings, but to highlight the fact that similar movement characteristics were used during the posttest. Additional information about this study has been included within this section to provide clarity in our revised manuscript.

Method
• It is unclear why three distances were used for training (ln 180-181)? Did participants do the same number of putts at each distance, or did they choose where to putt from? Did the experimenters control for the potential of a recency effect of practicing at the 1.83 distance when the pre-test and post-test were just done at 1.83?

**Response**: A serial practice design was used to enhance the motor learning effects from both practice groups, based on a significant amount of motor learning research showing that variable serial practice (i.e., a,b,c…) results in larger motor learning effects compared to constant practice (i.e., a,a,a…). The same number of putts were performed at each distance and participants were not provided a choice. This has been included within the method section.

• The procedure of how kinematic data was measured is missing from the method section and should be provided for replicability purposes.

**Response**: A digital camera, with a capture rate of 60 hz, was placed perpendicular to the participant at a distance of two-meters. The digital camera recorded the action of the golf putt. Dartfish software was used to measure the distance of the backswing and follow-through displacement. The time-course measure in the Dartfish software was used to determine the amount of time it took participants to complete each putt. Golf club head velocity was calculated by determining how quickly the club head moved through the range of motion from the maximum backswing position to ball contact for each putt. This information has been added to the revised manuscript.

Figures and Tables
• The figure caption for Figure 1 suggests it is for general procedure but includes phrase "Delayed Transfer" under Test 3 which only relates to the RW condition and not the VR condition - either the figure or the caption should be adjusted to more accurately present what is being shown.

**Response**: The titles within the figures have been adjusted to “immediate posttest” and “delayed posttest” to more accurately present the information. This clarification has also been included within the procedure.

Results, Discussion & Conclusions:
• Pairwise comparisons were included in the results, but an explanation was not posited for the differences observed. Would be beneficial for these differences to be addressed (i.e. For accuracy scores, why both test 3 and 4 for significantly lower than test 1 but test 2 was not; would it both be expected that the immediate post-test would also demonstrate improvement of scores after the training session? Do the authors have a possible explanation for this pattern of results?)

**Response**: Thank you for this recommendation. An explanation has been provided in the results section. See paragraph 5 in the discussion section.

• Without the specifics of how kinematic data was measured, it is difficult to interpret the results provided.

**Response**: We have added details to the revised Method section explaining how kinematics were measured.

• In line 239 the authors provide a non-significant p-value of p = 0.051 and use this value as support for no between-group differences in club velocity. However, given the very close proximity to significance, and the lack of a power analysis provided, this value should be addressed in the discussion as what could be a possible group difference in larger samples and the implications.

**Response**: This has been included within the discussion section. Specifically, it was included within the newly added limitation section.

• It would help to have the reported implications addressed in terms of the theoretical framework discussed in the introduction of the manuscript. Specifically, how do the results align (or not align) with the identical elements theory, identical production model and/or transfer appropriate processing theory? Do these theories offer potential explanation for why the observed results occurred?

**Response**: Thank you for this recommendation. A section has been added in the discussion section.

• Limitations to the study design should be included in the discussion.

**Response**: The limitations of the present study have been included within the discussion section.

6. PLOS authors have the option to publish the peer review history of their article ([what does this mean?](https://journals.plos.org/plosone/s/editorial-and-peer-review-process#loc-peer-review-history)). If published, this will include your full peer review and any attached files.


If you choose “no”, your identity will remain anonymous but your review may still be made public.


**Do you want your identity to be public for this peer review?** For information about this choice, including consent withdrawal, please see our [Privacy Policy](https://www.plos.org/privacy-policy).

Reviewer #1: No

Reviewer #2: No

[NOTE: If reviewer comments were submitted as an attachment file, they will be attached to this email and accessible via the submission site. Please log into your account, locate the manuscript record, and check for the action link "View Attachments". If this link does not appear, there are no attachment files.]

While revising your submission, please upload your figure files to the Preflight Analysis and Conversion Engine (PACE) digital diagnostic tool, <https://pacev2.apexcovantage.com/>. PACE helps ensure that figures meet PLOS requirements. To use PACE, you must first register as a user. Registration is free. Then, login and navigate to the UPLOAD tab, where you will find detailed instructions on how to use the tool. If you encounter any issues or have any questions when using PACE, please email PLOS at [figures@plos.org](mailto:figures@plos.org). Please note that Supporting Information files do not need this step.
