# Supplementary material for: Off the shelf: Investigating transfer of learning using commercially available virtual reality equipment
Source: PLoS One. 2023 Oct 3;18(10):e0279856. doi: 10.1371/journal.pone.0279856 (PMC10547175; doi:10.1371/journal.pone.0279856)
Supplement: Attachment — Submitted filename: Responses to Reviewers.docx [file pone.0279856.s003.docx]

Editor Comments:
Both reviewers agreed that the author has done well responding to their comments.
Please briefly address the remaining few comments from reviewer #1. Specifically, address the need for longer-term follow-up in motor learning studies, and the applicability/practicality (or lack thereof) currently of using virtual reality training clinically.

**Response**: Thank you for the revision. The comments below, specifically from reviewer #1 have been address.


[Note: HTML markup is below. Please do not edit.]

Reviewers' comments:

Reviewer's Responses to Questions

**Comments to the Author**

1. If the authors have adequately addressed your comments raised in a previous round of review and you feel that this manuscript is now acceptable for publication, you may indicate that here to bypass the “Comments to the Author” section, enter your conflict of interest statement in the “Confidential to Editor” section, and submit your "Accept" recommendation.

Reviewer #1: All comments have been addressed

Reviewer #2: (No Response)

2. Is the manuscript technically sound, and do the data support the conclusions?

The manuscript must describe a technically sound piece of scientific research with data that supports the conclusions. Experiments must have been conducted rigorously, with appropriate controls, replication, and sample sizes. The conclusions must be drawn appropriately based on the data presented.

Reviewer #1: Yes

Reviewer #2: Yes

3. Has the statistical analysis been performed appropriately and rigorously?

Reviewer #1: Yes

Reviewer #2: Yes

4. Have the authors made all data underlying the findings in their manuscript fully available?

The [PLOS Data policy](http://www.plosone.org/static/policies.action#sharing) requires authors to make all data underlying the findings described in their manuscript fully available without restriction, with rare exception (please refer to the Data Availability Statement in the manuscript PDF file). The data should be provided as part of the manuscript or its supporting information, or deposited to a public repository. For example, in addition to summary statistics, the data points behind means, medians and variance measures should be available. If there are restrictions on publicly sharing data—e.g. participant privacy or use of data from a third party—those must be specified.

Reviewer #1: Yes

Reviewer #2: (No Response)

5. Is the manuscript presented in an intelligible fashion and written in standard English?

PLOS ONE does not copyedit accepted manuscripts, so the language in submitted articles must be clear, correct, and unambiguous. Any typographical or grammatical errors should be corrected at revision, so please note any specific errors here.

Reviewer #1: Yes

Reviewer #2: Yes

6. Review Comments to the Author

Please use the space provided to explain your answers to the questions above. You may also include additional comments for the author, including concerns about dual publication, research ethics, or publication ethics. (Please upload your review as an attachment if it exceeds 20,000 characters)

Reviewer #1: The authors provided viable and better-founded responses to all queries. In addition, they proposed a reorganization of writing and demonstrating the data with greater clarity.
For complete acceptance, I suggest that the authors place a study limitation related to the non-longitudinal follow-up (greater than one month follow-up) of the observed performance gains. Also that, new studies need to do a longer follow-up to verify greater gains in motor learning.
I also suggest placing a small paragraph in the discussion, related to the clinical applicability of training in virtual reality, taking into account why this training will be efficient.

After thet inclusions on discussion section, I think the article can be accept.

**Response**: Thank you for your feedback and comments. A limitation has been included within the discussion related to the non-longitudinal follow. Furthermore, additional information has been added in the discussion regarding the clinical applicability.

Reviewer #2: (No Response)

7. PLOS authors have the option to publish the peer review history of their article ([what does this mean?](https://journals.plos.org/plosone/s/editorial-and-peer-review-process#loc-peer-review-history)). If published, this will include your full peer review and any attached files.


If you choose “no”, your identity will remain anonymous but your review may still be made public.


**Do you want your identity to be public for this peer review?** For information about this choice, including consent withdrawal, please see our [Privacy Policy](https://www.plos.org/privacy-policy).

Reviewer #1: No

Reviewer #2: No
